# A Case of Metastatic Spinal Cord Compression in a Patient With Coexisting Chronic Lymphocytic Leukemia and Colon Cancer

**DOI:** 10.7759/cureus.97979

**Published:** 2025-11-27

**Authors:** Kasim Abbas, Sateesh Nagumantry

**Affiliations:** 1 Hematology, Peterborough City Hospital, Peterborough, GBR

**Keywords:** acalabrutinib, chronic lymphocytic leukaemia, colon cancer, dual malignancy, spinal cord compression

## Abstract

A 78-year-old patient with a known diagnosis of colorectal cancer and chronic lymphocytic leukemia (CLL) presented to the emergency department with progressive neurological symptoms and was found to have metastatic spinal cord compression (MSCC) on MRI. A CT-guided biopsy of the affected vertebral bodies confirmed the bone lesions to be of CLL origin, not colorectal. The patient was treated with radiotherapy and commenced on acalabrutinib, a tyrosine kinase inhibitor.

This case highlights the diagnostic and therapeutic considerations in managing spinal disease in a patient with coexisting hematological and solid organ malignancy. Multidisciplinary evaluation, timely biopsy, and individualized therapy are crucial in guiding treatment decisions for spinal cord compression in patients with dual malignancies.

## Introduction

Chronic lymphocytic leukemia (CLL) is a common hematological malignancy and is associated with an increased risk of secondary malignancies [1]. By itself, CLL is rarely the cause of bone lesions, and colorectal cancer is not known to metastasize to bone frequently [2]. Chronic lymphocytic leukemia is the most common form of adult leukemia; the median age of diagnosis is 70 years, and it is twice as common in males as in females [3]. It accounts for approximately 1% of all cancer cases in the UK [4].

Typically, CLL is an indolent disease, although clinical presentation varies between patients. There is often a combination of peripheral B-cell lymphocytosis, systemic symptoms, cytopenias, and the presence of lymphadenopathy/organomegaly [5]. Small lymphocytic lymphoma (SLL) is classified by the WHO to be the same disease as CLL, but with a varied manifestation, specifically the absence of peripheral blood lymphocytosis [5].

Involvement of bone in CLL is rare, with only a handful of case reports detailing such patients [2]. Metastatic spinal cord compression (MSCC) within the context of CLL is even more rare; in the few documented cases there are, it generally leads to poor outcomes [6]. Where bone lesions do occur in CLL, they are thought to be due to abnormal bone remodeling, resulting in osteolytic lesions and osteopenia [7]. It is also possible they may arise due to Richter’s transformation, a process in which CLL develops into an aggressive lymphoma, most commonly diffuse large B-cell lymphoma [8]. Richter’s transformation is thought to occur in 2% to 10% of CLL/SLL cases and presents with a rapid progression of symptoms and lymphadenopathy. Differentiation between CLL and Richter’s is made on histopathology findings, and presently, patients who undergo Richter's transformation have a poor prognosis due to a lack of effective treatment options [8]. Finally, if there are bone lesions in CLL, consideration should also be given to the possibility of metastasis from a coexisting primary malignancy. 

Colorectal cancer accounts for 11% of all new cancer cases in the UK and is the fourth most common cancer in the UK overall [9]. Metastasis to bone is more common than in CLL, but it is still an infrequent site of metastases. A systematic review of 29 studies concluded the incidence of bone metastases in metastatic colorectal cancer to be between 3% and 7% [10]. The incidence of spinal cord compression in patients with colorectal metastasis to the spine is not yet known. 

## Case presentation

A 74-year-old man was referred for colonoscopy under the two-week wait pathway after presenting with abdominal pain, altered bowel habits, and a positive fecal immunochemical test (FIT). The patient was fully active without restriction in his daily activities; therefore, baseline performance status was zero as per the Eastern Cooperative Oncology Group (ECOG) performance status scale. His only medical comorbidity was atrial fibrillation, for which he was taking edoxaban. Baseline full blood count (FBC) demonstrated a mild normocytic anemia with normal white cell count and no peripheral lymphocytosis (Table 1).

**Table 1:** Baseline FBC results FBC: Full blood count

Parameters	Patient values	Reference range
Haemoglobin (g/L)	125	130-180
Mean corpuscular volume (fL)	87	80-100
Mean corpuscular haemoglobin (pg)	30.4	27-33
Mean corpuscular haemoglobin concentration (g/dL)	34.9	32-36
Platelets (x 10^9^/L)	168	150-400
Total white cell count (x 10^9^/L)	4.9	4.0-11.0
Neutrophils (x 10^9^/L)	4.5	1.8-7.7
Lymphocytes (x 10^9^/L)	1.6	1.4-4.8

**Figure 1:**
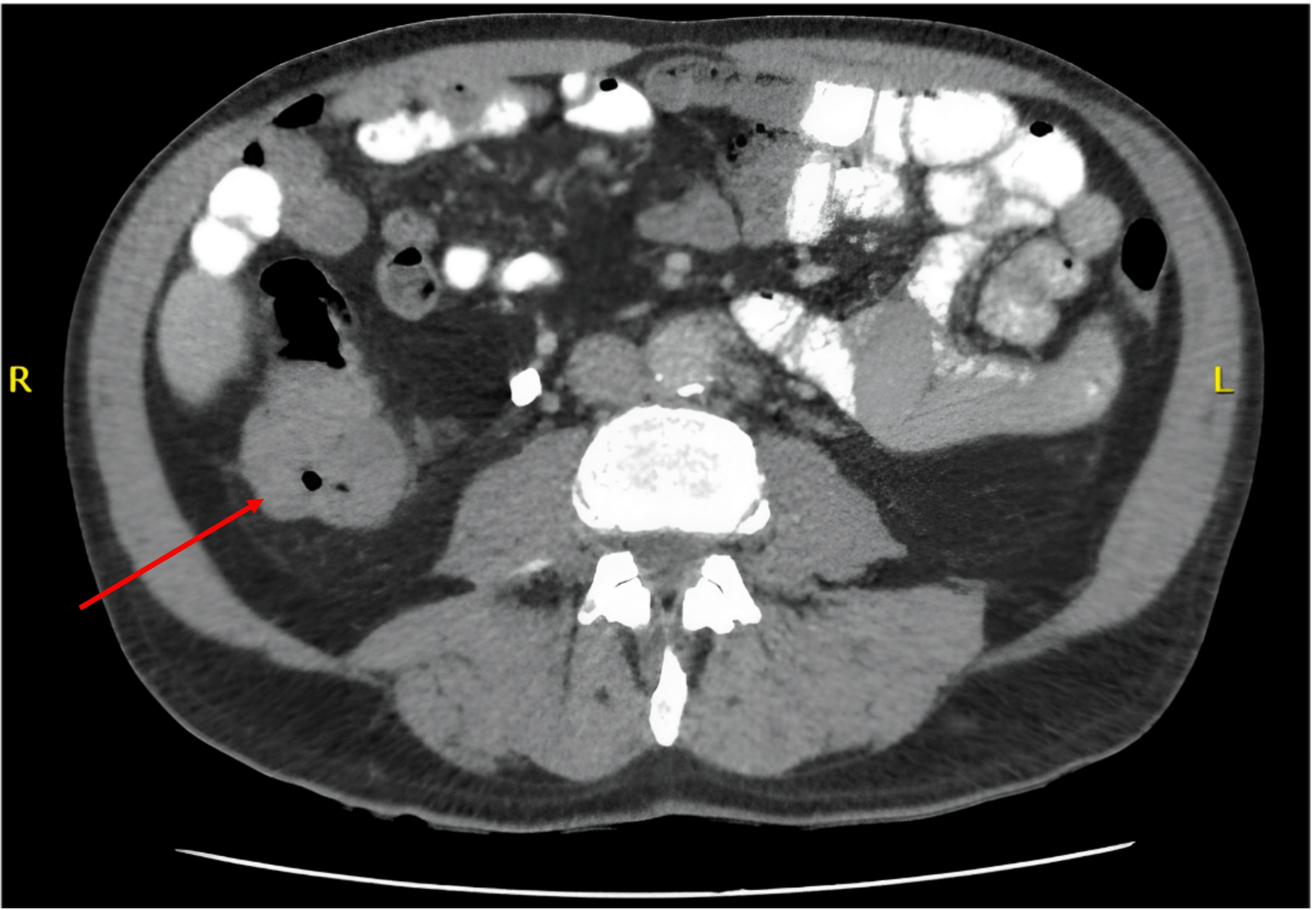
Axial view slice of contrast enhanced CT at the level of the hepatic flexure demonstrating a 6cm mass (red arrow) consistent with a primary colonic tumour

Colonoscopy identified a 6 cm lesion at the hepatic flexure of the ascending colon. Histological assessment demonstrated high-grade dysplasia without evidence of invasive malignancy. Staging CT of the chest, abdomen, and pelvis confirmed a 6 cm mass, a solitary 12 mm liver nodule, and several enlarged retroperitoneal lymph nodes (Figure 1).

**Figure 2:**
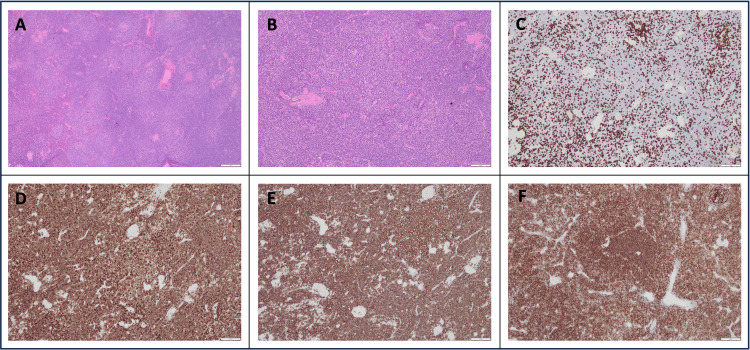
Immunohistochemistry of the abdominal lymph node sampling which confirmed CLL/SLL A: Hematoxylin and eosin stain at 4x magnification, B: Hematoxylin and eosin stain at 10x magnification, C: Biopsy stained for CD3 showing negative expression, D: Biopsy stained for CD5 showing positive expression, E: Biopsy stained for CD20 showing positive expression, F: Biopsy stained for CD23 showing positive expression

The patient underwent a right hemicolectomy with retroperitoneal lymph node sampling done at the same time. Histological analysis of the lymph nodes unexpectedly demonstrated features consistent with CLL/SLL (Figure 2). The sampled lymph nodes exhibited architectural effacement due to a lymphoid infiltrate with a pseudonodular pattern. Immunohistochemistry showed that the atypical cells were B lymphocytes expressing CD5 (Figure 2, panel D), CD20 (Figure 2, panel E), CD23 (Figure 2, panel F), BCL2, CD21, CD43, and LEF1, with a low proliferation index (<5%) as assessed by MIB1.

**Figure 3:**
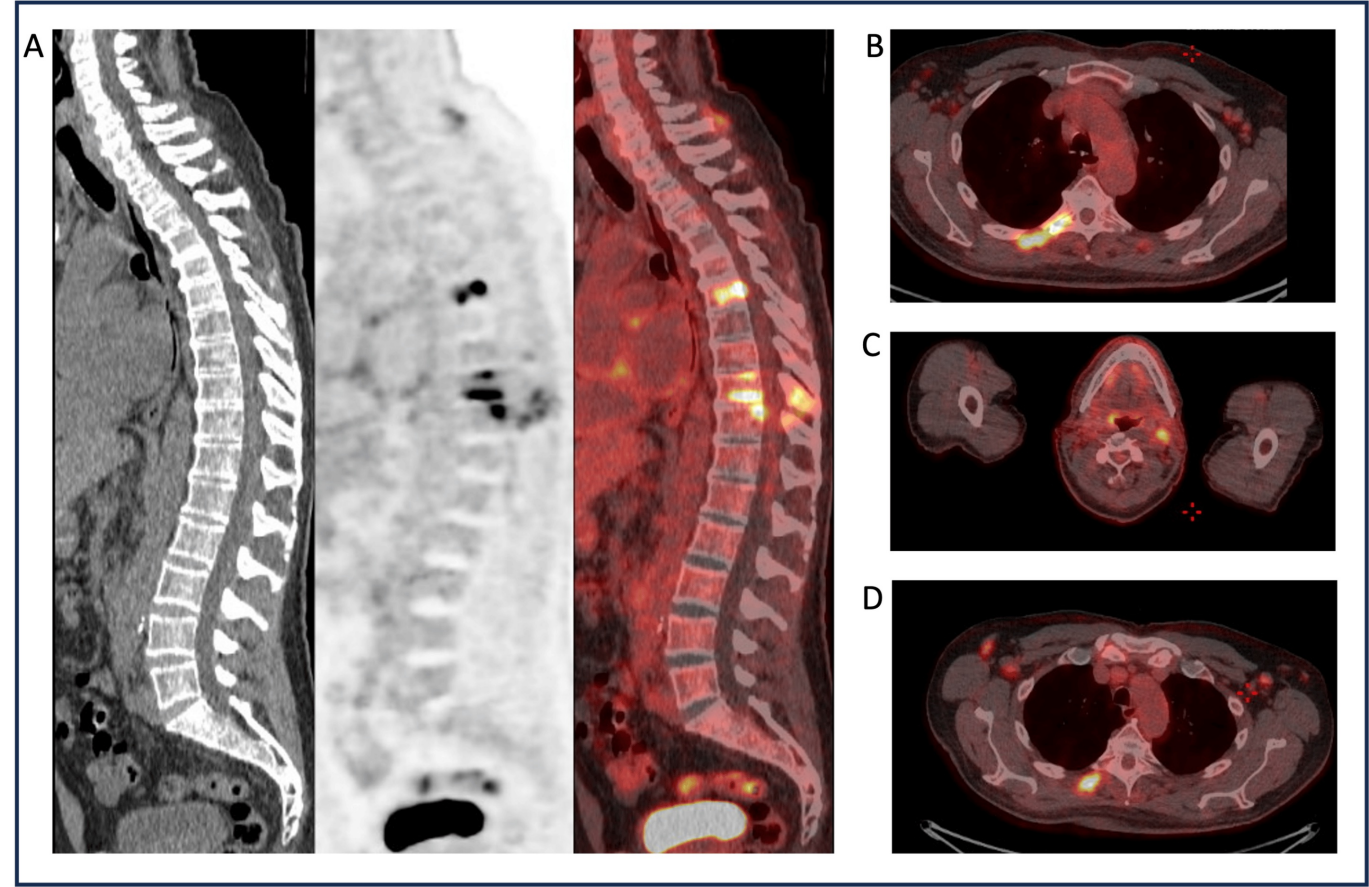
The FDG PET-CT images demonstrating widespread increased uptake in bone and lymph nodes A: Spinal series consisting of (from left to right) CT image, PET image, and fused PET-CT image in sagittal view, demonstrating increased uptake predominantly in thoracic vertebrae; B: Axial view of fused PET-CT image demonstrating increased uptake in ribs; C: Axial view of fused PET-CT image demonstrating increased uptake in cervical lymph nodes; D: Axial view of fused PET-CT image demonstrating increased uptake in axillary lymph nodes anteriorly and ribs posteriorly

Following surgery, the patient’s oncologist recommended deferring adjuvant chemotherapy pending further investigations. The patient recovered well from the surgery, maintained an ECOG performance status of zero, and did not report any new constitutional symptoms. Follow-up FBC post recovery from surgery was entirely normal, demonstrating a now normalized hemoglobin of >130 g/L and still no lymphocytosis. Lactate dehydrogenase (LDH) was within the normal range at 239 IU/L (<250 IU/L), as were serum electrophoresis and β2-microglobulin. Fluorodeoxyglucose (FDG) positron emission tomography (PET) revealed multiple metabolically active bone lesions, predominantly in the vertebrae and ribs, and also widespread uptake in cervical, axillary, and inguinal lymph nodes (Figure 3). No metabolic uptake was identified in the liver lesion. A targeted bone marrow biopsy showed 15% lymphoma cell infiltration with a TP53 mutation, consistent with CLL/SLL.

**Figure 4:**
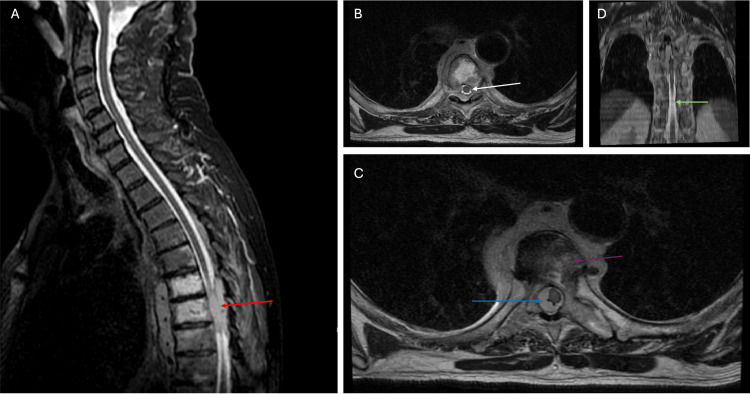
T2-weighted MRI images of the thoracic spine demonstrating pathological infiltration of the vertebrae and associated MSCC at the level of T6, T7, and T8 A: Sagittal T2-weighted MRI image showing an infiltrative lesion of the mid-thoracic vertebral bodies with extension into the posterior epidural space (red arrow). The mass results in marked effacement of the cerebrospinal fluid (CSF) space and flattening of the spinal cord. The heterogeneous T2 signal of the involved vertebral bodies is consistent with vertebral body infiltration from malignant disease. B: Axial T2-weighted MRI image at the level of compression demonstrating loss of a uniform bright CSF rim around the spinal cord (white arrow), in addition to a flattened and displaced spinal cord. C: Axial T2-weighted slice slightly inferior to panel B, showing soft tissue infiltration (blue arrow), continued deformation of the spinal cord, and loss of CSF space. Continued abnormal marrow signal within the vertebral body (purple arrow) is consistent with osseous involvement by malignant disease. D: Coronal T2-weighted MRI image allowing localisation of the lesion (green arrow). MSCC: Metastatic spinal cord compression

Repeat surveillance CT showed resolution of the liver lesion and again widespread lymphadenopathy. After a bone marrow biopsy and while awaiting the multidisciplinary team (MDT) outcome for the next stage of management, the patient presented to the emergency department with radicular back pain, weakness, and altered sensation in his legs. He was promptly assessed for neurological deficits, and urgent MRI imaging of the whole spine was arranged. On examination, there was symmetrical weakness in the lower limbs. The patient was able to weight bear, had a full range of motion, and there was movement against light resistance but not maximal resistance, consistent with a power grading of 4 out of 5 bilaterally as per the Medical Research Council (MRC) scale for muscle strength. Power in the upper limbs was 5 out of 5 on the MRC scale. On testing sensation, there was no sensory deficit or clear sensory level, and no perianal numbness. The MRI demonstrated new thoracic vertebral lesions with MSCC at the level of T6 to T8 (Figure 4).

The patient was initially managed with simple analgesia and started on high-dose oral corticosteroids. A single fraction of 8 Gy radiotherapy was delivered to T4-T10 within 24 hours of presentation. This was arranged following a neurosurgical consultation, which advised radiotherapy over immediate surgical intervention. As symptomatic compression was at the level of T6-T8, the radiotherapy field was focused on T4-T10, two vertebral levels below and above the level of compression, to minimize toxicity and treat only the areas with clinically significant tumor bulk. Radiotherapy was delivered prior to a vertebral biopsy being done, as the priority was urgent decompression to preserve neurological function. 

**Figure 5:**
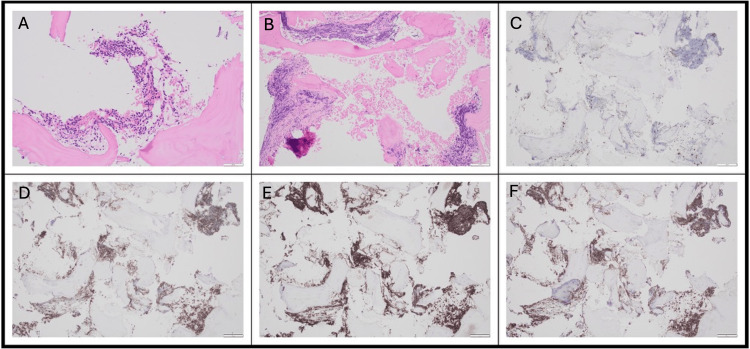
Histopathology slides of bone marrow trephine biopsy from T11 A and B: Hematoxylin and eosin stain at 10x magnification, C: Biopsy stained for CD3 showing negative expression, D: Biopsy stained for CD5 showing positive expression, E: Biopsy stained for CD20 showing positive expression, F: Biopsy stained for CD23 showing positive expression

With an established dual diagnosis of Dukes B colon cancer (T3N0) and CLL/SLL, uncertainty remained regarding the origin of the spinal disease. Solitary vertebral metastasis without other systemic recurrence would be an unusual presentation for colon cancer. Similarly, spinal cord compression due to early-stage CLL is rare. To clarify the etiology and guide potential treatment, a transpedicular biopsy of the T11 vertebral body was performed. T11 was chosen for the biopsy site as it had not been irradiated and was the safest vertebral level where diagnostic tissue could be obtained without risk of compromising spinal stability or neurological function. Biopsy demonstrated lymphoid infiltration with positive expression for B-cell markers (Figure 5), particularly CD5 (Figure 5, panel D), CD20 (Figure 5, panel E), and CD23 (Figure 5, panel F). Immunohistochemical findings were consistent with CLL, without evidence of additional pathology. The vertebral disease responsible for cord compression was considered a localized high-grade transformation of CLL, as non-transformed CLL rarely presents in this manner. 

At the hematology MDT meeting, it was decided to commence acalabrutinib with a low threshold for surveillance imaging. At this stage, the patient continued to experience residual back pain and required a walking frame for mobility. The MRI after three cycles of acalabrutinib demonstrated stable disease. Clinically, the patient had improved significantly, regaining independent mobility and the ability to drive. The consensus at MDT was to continue acalabrutinib with three-monthly hematology follow-up, alongside standard colorectal cancer surveillance with six-monthly CT scans.

## Discussion

Treatment options for MSCC may include a combination of corticosteroids, chemotherapy, radiotherapy, and surgery, with definitive management being either surgery or radiotherapy [11]. It is common practice for a decompressive laminectomy to be indicated if there is rapid neurological deterioration, pain has not responded to conservative measures, or the tumor is resistant to radiotherapy [11]. Radiation therapy for bone metastases is an effective measure at controlling pain in up to 80% of patients [2]. For our patient, his pain improved significantly post-radiotherapy. 

Management of this patient was aligned with the National Institute for Health and Care Excellence (NICE) 2023 NG234 guidelines for MSCC [12]. The guidelines state that in cases where immediate treatment is needed to prevent irreversible neurological loss, definitive treatment should not be delayed for histological confirmation. Therefore, this is why the patient had urgent radiotherapy before a biopsy of the vertebral body was obtained. When deciding between surgery and radiotherapy, NICE recommends radiotherapy if surgery is not suitable. Suitability for surgery is not defined by a single criterion; instead, it reflects multidisciplinary judgment to determine whether the patient is fit for major spinal surgery, has a sufficient overall prognosis to benefit, and has evidence of spinal instability requiring stabilization or progressive or severe neurological impairment [12].

The hospital to which our patient presented with spinal cord compression did not have an on-site neurosurgical team; therefore, an MDT decision was made with input from the nearest neurosurgical team in a separate hospital. As the patient had not had any previous radiotherapy, there was no evidence of spinal instability on imaging and no severe neurological deficits on examination. Radiotherapy was deemed more appropriate, as it could be arranged much quicker to manage his spinal cord compression and reassess his symptoms; he responded well and did not need any further intervention. 

The key step in determining ongoing management for our patient after his radiotherapy was obtaining a biopsy from the affected vertebral bodies. Clinically, it would be an unusual site of disease for both CLL and colon cancer, and the patient exhibited no systemic evidence of recurrence of his colon cancer or systemic symptoms suggestive of high-grade leukemia. A confirmed CLL/SLL origin of the vertebral lesions and exclusion of other pathology, particularly colorectal metastases or Richter's transformation, allowed discussion of the most appropriate therapy to initiate. The NICE guidelines on CLL management recommend monotherapy with ibrutinib or acalabrutinib, venetoclax with obinutuzumab, or idelalisib with rituximab in groups of patients with previously untreated CLL [13].

Up to one-third of patients with CLL will never require treatment for it. Indications to commence therapy include bulky or progressive lymphadenopathy or hepatosplenomegaly, cytopenias, the presence of ‘B’ symptoms such as drenching night sweats or weight loss, or rapid increase in white cell counts [14]. The patient in this case was commenced on therapy for CLL due to both progressive lymphadenopathy and, more importantly, his atypical presentation with spinal cord compression, a medical emergency.

Around 5% to 10% of CLL/SLL cases are considered high risk due to the presence of mutations such as TP53 and 17p deletion [1]. These mutations are associated with a poorer prognosis and increased risk of undergoing Richter’s transformation [15]. In particular, patients with TP53 mutations do not always respond well to chemo-immunotherapy. Therefore, they should be offered non-chemotherapy-based regimens that work on B-cell receptor pathways [16], as was the case with our patient, who demonstrated a good initial response to acalabrutinib. 

## Conclusions

This case emphasizes the clear diagnostic challenge in a patient with dual malignancies presenting with spinal cord compression and the crucial need for histologic confirmation of the source of metastasis for diagnosis and onward management. Assumptions based on disease prevalence can be misleading, especially in atypical patterns of metastases. The MDT discussions are of vital importance in formulating a personalized management plan for such patients. Coordination between both parent teams when there are dual competing diagnoses is crucial to ensure a patient-centered approach. Investigations should be done in a timely manner, and any treatments that may affect the outcomes should be withheld unless clinically urgent. This case serves as a reminder for clinicians to maintain a high index of suspicion for hematological involvement in atypical patterns of metastatic disease, especially if there is a previous history of hematological malignancy or lymphoproliferative disorders.

## References

[1] Shen Y, Coyle L, Kerridge I (2022). Second primary malignancies in chronic lymphocytic leukaemia: skin, solid organ, haematological and Richter's syndrome. EJ Haem.

[2] Hatoum G, Meshkin C, Alkhunaizi S, Levene R, Formoso-Onofrio J (2015). The risk of misdiagnosing the primary site responsible for bone metastases in patients with chronic lymphocytic leukemia and a second primary carcinoma. World J Oncol.

[3] Hallek M, Al-Sawaf O (2021). Chronic lymphocytic leukemia: 2022 update on diagnostic and therapeutic procedures. Am J Hematol.

[4] (2025). Chronic lymphocytic leukaemia (CLL) statistics | Cancer Research UK. https://www.cancerresearchuk.org/health-professional/cancer-statistics/statistics-by-cancer-type/leukaemia-cll.

[5] Santos FP, O'Brien S (2012). Small lymphocytic lymphoma and chronic lymphocytic leukemia: are they the same disease?. Cancer J.

[6] Majumdar G, Singh AK (1992). Cord compression: a rare complication of chronic lymphocytic leukaemia. J Clin Pathol.

[7] Prommer E (2004). Re: Chronic lymphocytic leukemia resembling metastatic bone disease. J Pain Symptom Manage.

[8] Wang Y, Sinha S, Wellik LE (2021). Distinct immune signatures in chronic lymphocytic leukemia and Richter syndrome. Blood Cancer J.

[9] (2025). Bowel cancer statistics | Cancer Research UK. https://www.cancerresearchuk.org/health-professional/cancer-statistics/statistics-by-cancer-type/bowel-cancer.

[10] Christensen TD, Jensen SG, Larsen FO, Nielsen DL (2018). Systematic review: incidence, risk factors, survival and treatment of bone metastases from colorectal cancer. J Bone Oncol.

[11] Younsi A, Riemann L, Scherer M, Unterberg A, Zweckberger K (2020). Impact of decompressive laminectomy on the functional outcome of patients with metastatic spinal cord compression and neurological impairment. Clin Exp Metastasis.

[12] (2025). Overview | spinal metastases and metastatic spinal cord compression | guidance | NICE. https://www.nice.org.uk/guidance/ng234.

[13] (2025). Overview | ibrutinib with venetoclax for untreated chronic lymphocytic leukaemia | Guidance | NICE. https://www.nice.org.uk/guidance/ta891.

[14] Shadman M (2023). Diagnosis and treatment of chronic lymphocytic leukemia: a review. JAMA.

[15] Zenz T, Eichhorst B, Busch R (2010). TP53 mutation and survival in chronic lymphocytic leukemia. J Clin Oncol.

[16] Shanafelt TD, Wang XV, Kay NE (2019). Ibrutinib-rituximab or chemoimmunotherapy for chronic lymphocytic leukemia. N Engl J Med.

